# Social vulnerability and survival among patients with high-grade glioma treated at a tertiary cancer center

**DOI:** 10.1007/s11060-026-05732-y

**Published:** 2026-07-30

**Authors:** Aileen H. Hsi, Chetna Wathoo, Erick Campbell, Mohammed Nassif, Gregory M. Buchold, Lynne H. Nguyen, Lorna H. McNeill, Ernest Hawk, Vinay K. Puduvalli, Carlos Kamiya-Matsuoka

**Affiliations:** 1 Department of Neuro-Oncology, Division of Cancer Medicine, The University of Texas MD Anderson Cancer Center, Houston, TX 77030 USA; 2 Department of Health Disparities Research, Division of Cancer Prevention and Population Sciences, The University of Texas MD Anderson Cancer Center, Houston, TX USA; 3 Division of Cancer Prevention and Population Sciences, The University of Texas MD Anderson Cancer Center, Houston, TX USA

**Keywords:** Glioblastoma, IDH-mutant grade 4 astrocytoma, Social Vulnerability Index, Overall Survival, Health disparities

## Abstract

**Purpose:**

We examined associations of county- and tract-level Social Vulnerability Index (SVI) with survival and treatment-related outcomes in adults with IDH-wildtype glioblastoma (GBM) or IDH-mutant grade 4 astrocytoma.

**Methods:**

Adults treated at MD Anderson from 2020 to 2025 with PROACTIVE consent, survival follow-up, and Texas residential data were included. The 2022 CDC/ATSDR SVI was modeled per 0.1 increase (10 percentile points). Primary Cox models for GBM adjusted for age, sex, performance status, extent of resection, MGMT methylation, insurance, and distance. Proportional hazards were assessed using Grambsch–Therneau tests.

**Results:**

Cohorts included 398 patients with GBM and 62 with IDH-mutant grade 4 astrocytoma. In GBM, SVI was not associated with survival in county-level analyses (HR per 0.1 increase, 1.00; 95% CI, 0.96–1.05; *P* = 0.920) or Houston-area tract-level analyses (HR, 1.05; 95% CI, 0.98–1.13; *P* = 0.176). In exploratory time-varying analysis, the SVI–mortality association attenuated over follow-up (interaction *P* = 0.028). Higher tract-level SVI was associated with greater odds of not undergoing gross-total resection (OR, 1.19; 95% CI, 1.07–1.33; q = 0.010) and lower odds of clinical-trial treatment (OR, 0.81; 95% CI, 0.71–0.92; q = 0.008). No significant survival association was identified in exploratory astrocytoma analyses.

**Conclusion:**

Among patients who reached tertiary neuro-oncology care, primary Cox models did not identify a statistically significant association between SVI and survival. Higher tract-level SVI was associated with less extensive resection and lower odds of clinical-trial treatment.

**Supplementary Information:**

The online version contains supplementary material available at https://doi.org/10.1007/s11060-026-05732-y.

## Introduction

 High-grade gliomas (HGGs), including glioblastoma, IDH-wildtype, CNS WHO grade 4 (GBM) and astrocytoma, IDH-mutant, CNS WHO grade 4, remain lethal despite surgery, radiotherapy, and temozolomide [1–4]. Prognosis is shaped by performance status, extent of resection, and MGMT promoter methylation [5, 6], but these factors do not capture social and structural barriers to specialized care.

Sociodemographic disadvantage and nonprivate insurance have been associated with lower rates of gross total resection (GTR), chemoradiotherapy, and timely radiotherapy in GBM [7–10]. Treatment at academic, high-volume, or specialized centers has also been associated with greater use of multimodal therapy and longer survival [11–13].

The CDC/ATSDR Social Vulnerability Index (SVI) is an area-level measure across four domains: socioeconomic status, household characteristics/disability, racial and ethnic minority status/language, and housing/transportation [14, 15]. Oncology studies have examined SVI in relation to screening, incidence, mortality, and other outcomes [16, 17], but its association with HGG outcomes among patients who reach tertiary care remains uncertain.

County-level SVI is widely available but may obscure neighborhood variation; tract-level SVI provides finer geographic resolution [14, 15].

We examined county- and tract-level SVI in relation to overall survival (OS) among adults with GBM or IDH-mutant grade 4 astrocytoma treated at MD Anderson. We also examined associations with insurance, performance status, extent of resection, upfront radiotherapy plus temozolomide, and clinical-trial treatment.

## Methods

### Study design and population

We conducted a retrospective cohort study of adults aged 18 years or older with HGG treated at The University of Texas MD Anderson Cancer Center from 2020 to 2025 who provided PROACTIVE consent for research use of clinical data. Patients were identified using ICD-10 codes and manually reviewed datasets. Eligible tumors were GBM and IDH-mutant grade 4 astrocytoma, based on available histopathologic and molecular data. We excluded patients without survival follow-up, sufficient Texas residential information for SVI assignment, or adequate pathologic or molecular data to confirm eligibility. The available extracts lacked a complete nonconsented registry and sufficient information to apply the same eligibility criteria, precluding reliable quantification of otherwise eligible patients without PROACTIVE consent.

The county-level cohorts included 398 patients with GBM and 62 with IDH-mutant grade 4 astrocytoma. The prespecified Houston area comprised Austin, Brazoria, Chambers, Fort Bend, Galveston, Harris, Liberty, Montgomery, and Waller counties. This subset included 263 patients with GBM and 39 with IDH-mutant grade 4 astrocytoma; complete tract-level SVI and all four domain scores were available for 260 and 39 patients, respectively. The MD Anderson Institutional Review Board approved the study.

### SVI assignment

County- and tract-level values were obtained from the 2022 CDC/ATSDR Texas SVI database. County SVI was assigned by county of residence; geocoded addresses were linked to census tract GEOIDs to assign tract-level SVI.

SVI values are percentile rankings from 0 to 1, with higher values indicating greater vulnerability. Overall SVI was the primary exposure; the socioeconomic status, household characteristics/disability, racial and ethnic minority status/language, and housing/transportation domains were secondary exposures. SVI was modeled per 0.1 increase, corresponding to 10 percentile points, and as fixed groups: <0.25, 0.25–0.49, 0.50–0.74, and ≥ 0.75.

### Outcomes, covariates, and statistical analyses

The primary outcome was OS from diagnosis to death from any cause or last follow-up. Patients alive at last contact were censored on that date. Survival time was converted from days to months using 30.4375 days per month; median follow-up was summarized among patients alive at last contact.

Proportional-hazards assumptions were evaluated with term-specific and global Grambsch–Therneau score tests based on Schoenfeld residuals and a left-continuous Kaplan–Meier time transformation.

Primary adjusted GBM Cox models included age, sex, KPS, extent of resection, MGMT promoter methylation, insurance, and distance to MD Anderson. Age and distance were continuous; KPS was categorized as ≥ 70 or < 70. Extent of resection was classified as GTR, near-GTR, subtotal resection, biopsy, or unknown.

The GBM MGMT variable distinguished methylated from combined unmethylated or unknown/not documented status because the latter categories could not be separated reliably. Insurance was reported as private/commercial, Medicare, Medicaid, self-pay/uninsured, other, or unknown/missing. The source field combined self-pay and uninsured status. Because nonprivate categories were small, models compared private/commercial with all remaining categories; a sensitivity analysis excluded other or unknown insurance.

Astrocytoma analyses were exploratory because of the smaller cohort and fewer events and included fixed county-SVI groups, age- and insurance-stratified analyses, and interaction tests.

Baseline characteristics were compared across fixed SVI groups using Kruskal–Wallis, chi-square, or Fisher exact tests. Kaplan–Meier methods estimated survival, log-rank tests compared regional distributions, and Cox models estimated SVI–OS associations. Multivariable models used complete cases. Benjamini–Hochberg false discovery rate correction was applied within each prespecified domain or treatment-outcome family.

Sequential Cox models assessed changes in the SVI estimate as covariates were added. Upfront radiotherapy plus temozolomide was defined as radiotherapy with concurrent and/or adjuvant temozolomide after the index grade 4 diagnosis. An exploratory model additionally included this variable and clinical-trial treatment; because both may reflect downstream eligibility and treatment selection after tertiary-center entry, it was not primary.

Age- and sex-adjusted logistic models examined associations of overall and domain-specific SVI with nonprivate insurance, KPS < 70, resection less extensive than GTR, upfront radiotherapy plus temozolomide, and clinical-trial treatment. Patients with unknown extent of resection were excluded from the less-than-GTR analysis.

Standardized perioperative steroid, comorbidity, and household travel-burden data were unavailable. Distance to MD Anderson was included as a geographic measure but not interpreted as household travel burden.

Secondary analyses compared the prespecified Houston area with the remainder of Texas. All tests were two-sided. Analyses were performed using Python.

## Results

### Cohort characteristics

Cohort characteristics are summarized in Table 1, and analytic cohorts in Table 2. The GBM cohort included 398 patients; median age was 60.5 years, 152 (38.2%) were female, and 374 of 396 patients (94.4%) with available KPS had a score of 70 or higher. MGMT promoter methylation was documented in 185 patients (46.5%). Upfront radiotherapy plus temozolomide and clinical-trial treatment were documented in 378 (95.0%) and 115 (28.9%), respectively. At last follow-up, 254 patients (63.8%) had died. Median OS was 22.7 months, and median follow-up among 144 patients alive was 23.5 months (IQR, 12.2–38.0).

**Table 1:** Patient characteristics, SVI, and selected clinical features by analytic cohort^a^

Characteristic	GBM (*n* = 398)	IDH-mutant grade 4 astrocytoma (*n* = 62)^b^
**A. Cohort, survival follow-up, and SVI linkage**		
Age at diagnosis, median (IQR), years	60.5 (51.0–68.0)	35.0 (27.0-42.8)
Female sex, n (%)	152 (38.2%)	25 (40.3%)
Deaths, n (%)	254 (63.8%)	23 (37.1%)
Alive/censored, n (%)	144 (36.2%)	39 (62.9%)
Median OS, months	22.7	147.6
Median follow-up among patients alive at last contact, months (IQR)^c^	23.5 (12.2–38.0)	61.1 (23.7-130.6)
County-level SVI available, n	398	62
County-level SVI, median (IQR)	0.619 (0.166–0.834)	0.834 (0.518–0.834)
County-level SVI, range	0.040–0.949	0.047–0.929
Houston-area county subset, n	263	39
Tract-level SVI available, n	260	39
Tract-level SVI, median (IQR)	0.229 (0.103–0.444)	0.249 (0.135–0.376)
Tract-level SVI, range	0.001–0.971	0.001–0.874
**B. Clinical**,** molecular**,** insurance**,** and treatment characteristics**		
Insurance status, n (%)		
Private/commercial	222 (55.8%)	40 (64.5%)
Medicare	155 (38.9%)	17 (27.4%)
Medicaid	5 (1.3%)	2 (3.2%)
Self-pay/uninsured	5 (1.3%)	0
Other	10 (2.5%)	3 (4.8%)
Unknown/missing	1 (0.3%)	0
KPS at diagnosis available, n^c^	396	62
KPS ≥ 70, n/N (%)	374/396 (94.4%)	61/62 (98.4%)
KPS < 70, n/N (%)	22/396 (5.6%)	1/62 (1.6%)
Extent of resection, n (%)		
GTR	189 (47.5%)	30 (48.4%)
Near-GTR	22 (5.5%)	6 (9.7%)
STR	103 (25.9%)	22 (35.5%)
Biopsy	65 (16.3%)	4 (6.5%)
Unknown	19 (4.8%)	0
MGMT promoter methylation, n (%)		
Methylated	185 (46.5%)	18 (29.0%)
Unmethylated or unknown/not documented	213 (53.5%)	19 (30.6%) unmethylated 25 (40.3%) unknown/not documented
Upfront radiotherapy plus temozolomide, n (%)		
Yes	378 (95.0%)	58 (93.5%)
No	19 (4.8%)	4 (6.5%)
Unknown	1 (0.3%)	0
Treatment on a clinical trial, n (%)		
Yes	115 (28.9%)	8 (12.9%)
No	283 (71.1%)	54 (87.1%)

Abbreviations: GBM, IDH-wildtype glioblastoma; GTR, gross total resection; IQR, interquartile range; KPS, Karnofsky Performance Status; MGMT, O6-methylguanine-DNA methyltransferase; OS, overall survival; STR, subtotal resection; SVI, Social Vulnerability Index

a Percentages may not total 100 because of rounding. Upfront radiotherapy plus temozolomide was defined as radiotherapy with concurrent and/or adjuvant temozolomide after the index grade 4 diagnosis. Treatment on a clinical trial refers to documented trial therapy after the index grade 4 diagnosis. For GBM, unmethylated or unknown/not documented category combines these statuses because these could not be separated reliably. The source insurance field combined self-pay and uninsured status

^b^ The astrocytoma cohort included patients with astrocytoma, IDH-mutant, CNS WHO grade 4

c KPS percentages use the number of patients with available KPS at diagnosis. Median follow-up is reported among patients alive at last contact

**Table 2:** Analytic cohorts for county- and census tract-level SVI analyses

Analysis cohort	GBM	IDH-mutant grade 4 astrocytoma
County-level analytic cohort	*n* = 398; 254 deaths	*n* = 62; 23 deaths
Primary county Cox cohort	*n* = 395; 251 deaths	Exploratory SVI-only, *n* = 62; 23 deaths
Houston-area county subset	*n* = 263	*n* = 39
Tract-level SVI cohort	*n* = 260; 157 deaths	*n* = 39; 11 deaths

County analyses used Texas residential county data. Tract analyses were restricted to the prespecified nine-county Houston-area geocoded subset. GBM multivariable Cox models used complete-case analysis. Astrocytoma analyses were exploratory because of the smaller cohort and fewer events

County-level SVI was available for all 398 patients with GBM (median, 0.619; IQR, 0.166–0.834; range, 0.040–0.949). The Houston-area subset included 263 patients; 260 had complete tract-level SVI and domain scores, with 157 deaths. Median tract-level SVI was 0.229 (IQR, 0.103–0.444; range, 0.001–0.971).

The astrocytoma cohort included 62 patients; median age was 35.0 years, 25 (40.3%) were female, and 23 (37.1%) had died. MGMT status was methylated in 18 (29.0%), unmethylated in 19 (30.6%), and unknown/not documented in 25 (40.3%). Upfront radiotherapy plus temozolomide and clinical-trial treatment were documented in 58 (93.5%) and 8 (12.9%), respectively. Median OS was 147.6 months, and median follow-up among 39 patients alive was 61.1 months (IQR, 23.7–130.6). Median county SVI was 0.834 (IQR, 0.518–0.834; range, 0.047–0.929). The tract cohort included 39 patients and 11 deaths; median tract SVI was 0.249 (IQR, 0.135–0.376; range, 0.001–0.874).

### SVI and overall survival

In the primary adjusted GBM model, the HR per 0.1 increase in county SVI was 1.00 (95% CI, 0.96–1.05; *P* = 0.920; *n* = 395, 251 deaths; Table 3). The global test across fixed SVI groups yielded *P* = 0.999. The SVI-specific proportional-hazards test yielded *P* = 0.162, whereas the global test yielded *P* = 0.016, suggesting nonproportionality in another model term. A model stratified by insurance and extent of resection yielded a similar estimate (HR, 1.01; 95% CI, 0.96–1.06; *P* = 0.715).

**Table 3:** Cox models for SVI and overall survival

Cohort	SVI scale	SVI measure	*n*	Deaths	HR	95% CI	*P* value
GBM	County	Per 0.1 increase	395	251	1.00	0.96–1.05	0.920
GBM	County	Group 2 vs. group 1	395	251	1.03	0.65–1.62	0.915
GBM	County	Group 3 vs. group 1	395	251	1.00	0.66–1.52	0.985
GBM	County	Group 4 vs. group 1	395	251	1.02	0.74–1.42	0.890
GBM	Census tract	Per 0.1 increase	260	157	1.05	0.98–1.13	0.176
GBM	Census tract	Group 2 vs. group 1	260	157	0.98	0.66–1.44	0.906
GBM	Census tract	Group 3 vs. group 1	260	157	1.89	1.16–3.07	0.010
GBM	Census tract	Group 4 vs. group 1	260	157	0.93	0.40–2.17	0.869
Astrocytoma	County	Per 0.1 increase	62	23	1.04	0.89–1.21	0.643
Astrocytoma	Census tract	Per 0.1 increase	39	11	1.12	0.85–1.49	0.422

Abbreviations: CI, confidence interval; GBM, IDH-wildtype glioblastoma; HR, hazard ratio; SVI, Social Vulnerability Index

^a^ GBM primary models adjusted for age at diagnosis, sex, KPS at diagnosis, five-category extent of resection, MGMT promoter methylation status, insurance status, and distance to MD Anderson. Astrocytoma analyses used exploratory SVI-only models

^b^ Fixed SVI groups were < 0.25, 0.25–0.49, 0.50–0.74, and ≥ 0.75, with the lowest group as the reference. For GBM, the county group global P value was 0.999; the tract group global P value was 0.052, and the ordinal-trend P value was 0.191

In the Houston-area GBM tract cohort, the primary adjusted HR was 1.05 per 0.1 increase in SVI (95% CI, 0.98–1.13; *P* = 0.176; *n* = 260, 157 deaths; Table 3). The fixed-group pattern was nonmonotonic: group 3 had a higher hazard than group 1 (HR, 1.89; 95% CI, 1.16–3.07; *P* = 0.010), whereas groups 2 and 4 were not significantly different from group 1. The global and ordinal-trend tests yielded *P* = 0.052 and *P* = 0.191, respectively. The SVI-specific and global proportional-hazards tests yielded *P* = 0.051 and *P* = 0.145.

The county-level SVI HR remained close to 1.00 across sequential models. In the tract cohort, the HR was 1.00 in the SVI-only model, 1.05 in the primary model, and 1.07 after adding upfront radiotherapy plus temozolomide and clinical-trial treatment (95% CI, 0.99–1.15; *P* = 0.087). In the treatment-adjusted model, the SVI-specific and global proportional-hazards test P values were 0.066 and 0.090 (Online Resource 1, Supplementary Tables 1 and 6).

In an exploratory time-varying model, the SVI-by-log-time interaction yielded *P* = 0.028. Estimated HRs per 0.1 increase were 1.17 (95% CI, 1.04–1.32) at 6 months, 1.09 (95% CI, 1.01–1.17) at 12 months, and 1.01 (95% CI, 0.92–1.10) at 24 months. An unplanned piecewise analysis estimated HRs of 1.24 before 12 months and 0.96 thereafter and was considered exploratory. Insurance sensitivity analyses yielded similar estimates (Online Resource 1, Supplementary Tables 6 and 7).

In exploratory astrocytoma analyses, county- and tract-level HRs were 1.04 (95% CI, 0.89–1.21; *P* = 0.643; *n* = 62, 23 deaths) and 1.12 (95% CI, 0.85–1.49; *P* = 0.422; *n* = 39, 11 deaths), respectively. Proportional-hazards test P values were 0.661 and 0.505. Age- and insurance-stratified HRs ranged from 1.02 to 1.14, without evidence of interaction by age (*P* = 0.653) or insurance (*P* = 0.302). Median OS across fixed county-SVI groups was 189.5, 19.5, 173.6, and 147.6 months, without a monotonic pattern. Because of tied values, small groups, and few events, these estimates were descriptive (Online Resource 1, Supplementary Table 5).

### SVI and treatment-related outcomes

In age- and sex-adjusted analyses of the Houston-area GBM tract cohort (Table 4), overall SVI was associated with nonprivate insurance before but not after FDR correction (OR per 0.1 increase, 1.14; 95% CI, 1.01–1.29; *P* = 0.038; q = 0.093). The housing/transportation domain remained associated after correction (OR, 1.16; 95% CI, 1.04–1.29; *P* = 0.007; q = 0.037).

**Table 4:** Selected treatment-related outcomes in the Houston-area GBM tract cohort

Outcome	SVI measure	*n*	Outcome present, *n*	OR per 0.1	95% CI	*P* value	FDR q
Nonprivate insurance	Overall SVI	260	125	1.14	1.01–1.29	0.038	0.093
Nonprivate insurance	Housing/transportation	260	125	1.16	1.04–1.29	0.007	0.037
Less than GTR	Overall SVI	251	117	1.19	1.07–1.33	0.002	0.010
Less than GTR	Socioeconomic status	251	117	1.18	1.05–1.31	0.004	0.010
Less than GTR	Housing/transportation	251	117	1.11	1.01–1.22	0.024	0.039
Clinical trial treatment	Overall SVI	260	80	0.81	0.71–0.92	0.002	0.008
Clinical trial treatment	Socioeconomic status	260	80	0.84	0.74–0.95	0.006	0.016
Clinical trial treatment	Minority status/language	260	80	0.87	0.77–0.99	0.033	0.042
Clinical trial treatment	Housing/transportation	260	80	0.87	0.78–0.97	0.010	0.016
Upfront RT plus TMZ	Overall SVI	260	247	0.91	0.72–1.15	0.420	0.675
KPS < 70	Overall SVI	260	14	0.97	0.76–1.23	0.811	0.947

Abbreviations: CI, confidence interval; FDR, false discovery rate; GBM, IDH-wildtype glioblastoma; GTR, gross total resection; KPS, Karnofsky Performance Status; OR, odds ratio; RT, radiotherapy; SVI, Social Vulnerability Index; TMZ, temozolomide

^a^ Odds ratios are reported per 0.1-unit increase in SVI and were adjusted for age and sex. Benjamini–Hochberg FDR correction was applied separately within each outcome family. Patients with unknown extent of resection were excluded from the less-than-GTR analysis

Higher overall tract SVI was associated with resection less extensive than GTR (OR, 1.19; 95% CI, 1.07–1.33; *P* = 0.002; q = 0.010). The socioeconomic and housing/transportation domains were also associated with this outcome (ORs, 1.18 and 1.11; q = 0.010 and 0.039).

Higher overall tract SVI was associated with lower odds of clinical-trial treatment (OR, 0.81; 95% CI, 0.71–0.92; *P* = 0.002; q = 0.008). The socioeconomic, minority status/language, and housing/transportation domains were also associated with lower trial-treatment odds (ORs, 0.84, 0.87, and 0.87; q = 0.016, 0.042, and 0.016). Overall tract SVI was not associated with upfront radiotherapy plus temozolomide or KPS < 70.

No county- or tract-level SVI domain was associated with GBM OS after FDR correction. Exploratory astrocytoma domain analyses were also null (Online Resource 1, Supplementary Table 3).

### Regional survival comparisons

Among patients with GBM, 263 Houston-area residents had 157 deaths and 135 patients elsewhere in Texas had 97 deaths. Median OS was 23.5 and 19.6 months, and 24-month OS was 49.4% and 44.1%, respectively (log-rank *P* = 0.006). The unadjusted HR for Houston-area residence was 0.70 (95% CI, 0.54–0.90; *P* = 0.006). These groups comprised all 398 patients and 254 deaths; pooled median OS was 22.7 months (Online Resource 1, Supplementary Table 2).

Among patients with IDH-mutant grade 4 astrocytoma, 39 Houston-area residents had 11 deaths and 23 patients elsewhere in Texas had 12 deaths. Median OS was not reached and 173.6 months, respectively; 24-month OS was 85.4% and 90.5% (log-rank *P* = 0.722). Regional analyses were exploratory (Online Resource 1, Supplementary Table 2).

## Discussion

In this single-center tertiary-care cohort, the primary Cox models did not identify a statistically significant association between county- or tract-level SVI and OS after adjustment for clinical, molecular, and access-related factors. An exploratory tract-level analysis suggested a stronger association early in follow-up that attenuated over time. Higher tract SVI was also associated with resection less extensive than GTR and lower odds of clinical-trial treatment. These associations are observational and do not establish causation.

The GBM median OS of 22.7 months exceeded many population-based estimates and the median in the original radiotherapy-plus-temozolomide trial [1, 4]. This may reflect referral and consent selection, favorable performance status and insurance coverage, contemporary molecular classification, frequent upfront radiotherapy plus temozolomide, and access to multidisciplinary care and trials. It should not be interpreted as evidence that treatment at our center caused longer survival.

The county-level GBM estimate was near the null with a narrow confidence interval, whereas the tract-level interval remained compatible with a modest adverse association. Fixed tract groups were nonmonotonic: only group 3 differed from group 1, with global *P* = 0.052 and ordinal-trend *P* = 0.191. The time-varying and piecewise models were exploratory. Thus, a modest adverse tract-level association, particularly early in follow-up, remains possible, but the nonmonotonic pattern and exploratory analyses limit firm conclusions.

The Houston-area comparison was descriptive. Longer survival among Houston-area patients may reflect referral patterns, proximity, clinical characteristics, or access to specialized treatment rather than an effect of residence.

Some disparities associated with social vulnerability may arise before tertiary referral. Patients in more vulnerable areas may face delays in diagnosis, referral, authorization, neurosurgical evaluation, or treatment, barriers to travel, or failure to reach a tertiary center. We lacked data on these intervals and on otherwise eligible patients who were never referred, so this possibility could not be tested directly. Requiring PROACTIVE consent may have introduced additional selection. The absence of a statistically significant association within this cohort does not exclude disparities before referral or among patients who never access tertiary care.

This retrospective, single-center study included only patients with PROACTIVE consent, sufficient Texas residential information, and tertiary neuro-oncology care. The number of otherwise eligible nonconsenting patients could not be determined. The low self-pay/uninsured proportion may reflect referral and consent selection. The source field combined self-pay and uninsured status, and sparse nonprivate categories required binary modeling. County SVI may obscure neighborhood variation, whereas tract analyses were limited to the nine-county Houston area. Astrocytoma analyses were exploratory because of the small cohort and few events.

The primary models adjusted for insurance, performance status, and extent of resection, which may lie partly on the pathway between social vulnerability and survival. Sequential models showed that the county estimate remained near the null, but formal mediation analysis was not possible; models with downstream variables estimate covariate-adjusted associations rather than the total causal effect. We lacked standardized perioperative steroid and comorbidity data and measures of travel costs, lodging, transportation, caregiver support, work disruption, and intervals from symptoms to diagnosis, referral, authorization, surgery, radiotherapy, or chemotherapy. Distance to MD Anderson was a geographic proxy, not household travel burden, and may be underestimated when temporary local addresses are recorded. SVI is an area-level measure and should not be interpreted as individual income, education, employment, language proficiency, transportation access, caregiver support, or other personal resources.

Among patients who reached tertiary neuro-oncology care, the primary Cox models did not identify a statistically significant association between area-level SVI and OS. Higher tract SVI was associated with less extensive resection and lower odds of clinical-trial treatment, and the exploratory time-varying analysis suggested a stronger early association that attenuated over time. These findings do not exclude disparities before referral or among patients who never reach tertiary care. Prospective multicenter studies combining individual socioeconomic and access measures with area-level SVI are needed to determine whether disparities arise before referral, during treatment, or both.

## Supplementary Information

Below is the link to the electronic supplementary material.


Supplementary Material 1: Online Resource 1. Supplementary Tables 1–7: sequential Cox models, regional survival comparisons, domain-specific analyses, availability of additional variables, astrocytoma subgroup analyses, proportional-hazards diagnostics and time-varying sensitivity analyses, and insurance-coding sensitivity analyses.


## Data Availability

The datasets generated and analyzed during the current study are not publicly available because they contain protected health information. They may be available from the corresponding author on reasonable request and with institutional approval.

## References

[1] Price M, Ballard CAP, Benedetti JR, Kruchko C, Barnholtz-Sloan JS, Ostrom QT (2025) CBTRUS statistical report: primary brain and other central nervous system tumors diagnosed in the United States in 2018–2022. Neuro Oncol 27(Suppl 4):iv1–iv66. 10.1093/neuonc/noaf194 41092086 PMC12527013

[2] Louis DN, Perry A, Wesseling P, Brat DJ, Cree IA, Figarella-Branger D et al (2021) The 2021 WHO classification of tumors of the central nervous system: a summary. Neuro Oncol 23:1231–1251. 10.1093/neuonc/noab106 34185076 PMC8328013

[3] Weller M, van den Bent M, Preusser M, Le Rhun E, Tonn JC, Minniti G et al (2021) EANO guidelines on the diagnosis and treatment of diffuse gliomas of adulthood. Nat Rev Clin Oncol 18:170–186. 10.1038/s41571-020-00447-z 33293629 PMC7904519

[4] Stupp R, Mason WP, van den Bent MJ, Weller M, Fisher B, Taphoorn MJB et al (2005) Radiotherapy plus concomitant and adjuvant temozolomide for glioblastoma. N Engl J Med 352:987–996. 10.1056/NEJMoa043330 15758009

[5] Hegi ME, Diserens AC, Gorlia T, Hamou MF, de Tribolet N, Weller M et al (2005) MGMT gene silencing and benefit from temozolomide in glioblastoma. N Engl J Med 352:997–1003. 10.1056/NEJMoa043331 15758010

[6] Molinaro AM, Hervey-Jumper S, Morshed RA, Young JS, Han SJ, Chunduru P et al (2020) Association of maximal extent of resection of contrast-enhanced and non-contrast-enhanced tumor with survival within molecular subgroups of patients with newly diagnosed glioblastoma. JAMA Oncol 6:495–503. 10.1001/jamaoncol.2019.6143 32027343 PMC7042822

[7] Dressler EV, Liu M, Garcia CR, Dolecek TA, Pittman T, Huang B et al (2019) Patterns and disparities of care in glioblastoma. Neurooncol Pract 6:37–46. 10.1093/nop/npy014 30740232 PMC6352755

[8] Estevez-Ordonez D, Abdelrashid M, Coffee E, Laskay NMB, Atchley TJ, Chkheidze R et al (2023) Racial and socioeconomic disparities in glioblastoma outcomes: a single-center retrospective cohort study. Cancer 129:3010–3022. 10.1002/cncr.34881 37246417

[9] Pollom EL, Fujimoto DK, Han SS, Harris JP, Tharin SA, Soltys SG et al (2018) Newly diagnosed glioblastoma: adverse socioeconomic factors correlate with delay in radiotherapy initiation and worse overall survival. J Radiat Res 59(Suppl 1):i11–i18. 10.1093/jrr/rrx103 29432548 PMC5868191

[10] Goyal A, Zreik J, Brown DA, Kerezoudis P, Habermann EB, Chaichana KL et al (2022) Disparities in access to surgery for glioblastoma multiforme at high-volume Commission on Cancer-accredited hospitals in the United States. J Neurosurg 137:32–41. 10.3171/2021.7.JNS211307 34767534

[11] Zhu P, Du XL, Zhu JJ, Esquenazi Y (2020) Improved survival of glioblastoma patients treated at academic and high-volume facilities: a hospital-based study from the National Cancer Database. J Neurosurg 132:491–502. 10.3171/2018.10.JNS182247 30771780

[12] Aulakh S, DeDeo MR, Free J, Rosenfeld SS, Quiñones-Hinojosa A, Paulus A et al (2019) Survival trends in glioblastoma and association with treating facility volume. J Clin Neurosci 68:271–274. 10.1016/j.jocn.2019.04.028 31133366 PMC8006067

[13] Raj R, Seppä K, Luostarinen T, Malila N, Seppälä M, Pitkäniemi J et al (2020) Disparities in glioblastoma survival by case volume: a nationwide observational study. J Neurooncol 147:361–370. 10.1007/s11060-020-03428-5 32060840 PMC7136186

[14] Flanagan BE, Gregory EW, Hallisey EJ, Heitgerd JL, Lewis B (2011) A social vulnerability index for disaster management. J Homel Secur Emerg Manag 8. 10.2202/1547-7355.1792

[15] Centers for Disease Control and Prevention/Agency for Toxic Substances and Disease Registry/Geospatial Research, Analysis, and Services Program (2022) CDC/ATSDR Social Vulnerability Index 2022 Database: Texas. https://www.atsdr.cdc.gov/place-health/php/svi/svi-data-documentation-download.html. Accessed 28 May 2026

[16] Tran T, Rousseau MA, Farris DP, Bauer C, Nelson KC, Doan HQ (2023) The social vulnerability index as a risk stratification tool for health disparity research in cancer patients: a scoping review. Cancer Causes Control 34:407–420. 10.1007/s10552-023-01683-1 37027053 PMC10080510

[17] Mehta A, Jeon WJ, Nagaraj G (2024) Association of US county-level social vulnerability index with breast, colorectal, and lung cancer screening, incidence, and mortality rates across US counties. Front Oncol 14:1422475. 10.3389/fonc.2024.1422475 39169944 PMC11335618

