# Supplementary material for: Social vulnerability and survival among patients with high-grade glioma treated at a tertiary cancer center
Source: J Neurooncol. 2026 Jul 30;179(1):21. doi: 10.1007/s11060-026-05732-y (PMC13424612; doi:10.1007/s11060-026-05732-y)
Supplement: Supplementary file 1 — Supplementary Material 1: Online Resource 1. Supplementary Tables 1–7: sequential Cox models, regional survival comparisons, domain-specific analyses, availability of additional variables, astrocytoma subgroup analyses, proportional-hazards diagnostics and time-varying sensitivity analyses, and insurance-coding sensitivity analyses. [file 11060_2026_5732_MOESM1_ESM.pdf]

Supplementary Information

Social vulnerability and survival among patients with high-grade glioma treated at a tertiary cancer center

Journal of Neuro-Oncology

Aileen H. Hsi, Chetna Wathoo, Erick Campbell, Mohammed Nassif, Gregory M. Buchold, Lynne H. Nguyen, Lorna H. McNeill, Ernest Hawk, Vinay K. Puduvalli, and Carlos Kamiya-Matsuoka

Corresponding author: Aileen H. Hsi, MD, MPH; ahhsi@mdanderson.org

Supplementary Table 1. Sequential Cox models for SVI and overall survival in GBM

SVI scale	Model	Covariates added	n	Deaths	HR per 0.1 SVI	95% CI	P value
County	Model 1	SVI only	398	254	0.99	0.95-1.03	0.571
County	Model 2	+ age and sex	398	254	1.00	0.96-1.04	0.821
County	Model 3	+ MGMT methylation	398	254	0.99	0.95-1.04	0.805
County	Model 4	+ KPS and EOR	396	252	0.99	0.95-1.03	0.619
County	Model 5 primary	+ insurance and distance	395	251	1.00	0.96-1.05	0.920
County	Model 6 exploratory	+ upfront RT/TMZ and trial treatment	395	251	1.00	0.95-1.04	0.872
Census tract	Model 1	SVI only	260	157	1.00	0.93-1.07	0.974
Census tract	Model 2	+ age and sex	260	157	1.05	0.98-1.13	0.186
Census tract	Model 3	+ MGMT methylation	260	157	1.05	0.98-1.13	0.175
Census tract	Model 4	+ KPS and EOR	260	157	1.03	0.96-1.11	0.359
Census tract	Model 5 primary	+ insurance and distance	260	157	1.05	0.98-1.13	0.176
Census tract	Model 6 exploratory	+ upfront RT/TMZ and trial treatment	260	157	1.07	0.99-1.15	0.087

Abbreviations: CI, confidence interval; GBM, IDH-wildtype glioblastoma; HR, hazard ratio; RT, radiotherapy; SVI, Social Vulnerability Index; TMZ, temozolomide.

[a] Model 5 was the primary full model and included age, sex, MGMT promoter methylation, KPS, five-category extent of resection, insurance, and distance to MD Anderson. Model 6 also included upfront radiotherapy plus temozolomide and treatment on a clinical trial and was exploratory because both variables may reflect downstream access, eligibility, and treatment selection.

Supplementary Table 2. Exploratory regional survival comparisons[a]

Cohort	Group	n	Deaths	Median OS, months	24-month OS	Log-rank P	Houston-area HR (95% CI)
GBM	Houston area	263	157	23.5	49.4%	0.006	0.70 (0.544-0.904)
GBM	Non-Houston	135	97	19.6	44.1%	0.006	
Astrocytoma	Houston area	39	11	Not reached	85.4%	0.722	0.86 (0.372-1.985)
Astrocytoma	Non-Houston	23	12	173.6	90.5%	0.722	

Abbreviations: CI, confidence interval; GBM, IDH-wildtype glioblastoma; HR, hazard ratio; OS, overall survival.

a These analyses were exploratory and were not part of the primary SVI survival models. Houston-area residence was defined by Austin, Brazoria, Chambers, Fort Bend, Galveston, Harris, Liberty, Montgomery, and Waller counties. The regional groups were mutually exclusive and comprised the full county-level cohort; OS was calculated from diagnosis using the same event and censoring definitions as the overall cohort.

[b] The unadjusted HR compares Houston-area with non-Houston residence.

[c] The astrocytoma cohort includes patients with astrocytoma, IDH-mutant, CNS WHO grade 4.

Supplementary Table 3. Domain-specific SVI survival analyses

Cohort/scale	Domain	n	Deaths	HR per 0.1	95% CI	P value	FDR q
GBM county	Socioeconomic status	395	251	1.00	0.96-1.04	0.989	0.989
GBM county	Household characteristics/disability	395	251	1.02	0.95-1.09	0.638	0.989
GBM county	Minority status/language	395	251	1.01	0.95-1.07	0.751	0.989
GBM county	Housing/transportation	395	251	1.00	0.94-1.07	0.936	0.989
Astrocytoma county	Socioeconomic status	62	23	1.02	0.89-1.17	0.761	0.761
Astrocytoma county	Household characteristics/disability	62	23	0.96	0.79-1.17	0.700	0.761
Astrocytoma county	Minority status/language	62	23	0.90	0.75-1.08	0.262	0.524
Astrocytoma county	Housing/transportation	62	23	1.12	0.92-1.37	0.249	0.524
GBM tract	Socioeconomic status	260	157	1.02	0.95-1.10	0.554	0.554
GBM tract	Household characteristics/disability	260	157	1.04	0.97-1.12	0.225	0.450
GBM tract	Minority status/language	260	157	1.03	0.96-1.11	0.359	0.478
GBM tract	Housing/transportation	260	157	1.04	0.98-1.11	0.198	0.450
Astrocytoma tract	Socioeconomic status	39	11	0.98	0.71-1.34	0.879	0.879
Astrocytoma tract	Household characteristics/disability	39	11	1.09	0.83-1.42	0.545	0.879
Astrocytoma tract	Minority status/language	39	11	1.03	0.78-1.35	0.841	0.879
Astrocytoma tract	Housing/transportation	39	11	1.21	0.95-1.54	0.126	0.505

Abbreviations: CI, confidence interval; FDR, false discovery rate; GBM, IDH-wildtype glioblastoma; HR, hazard ratio; SVI, Social Vulnerability Index. Effects are reported per 0.1-unit increase in domain SVI. Domain analyses were secondary, and Benjamini-Hochberg FDR correction was applied within each geographic-scale and cohort family.

Supplementary Table 4. Availability and handling of additional variables

Variable	Availability	How handled	Reason/limitation
PROACTIVE non-consent denominator	Not reliably reconstructable	Not calculated; addressed as a selection limitation	The available extracts did not include a complete non-consented registry or the information needed to apply the same diagnostic and molecular eligibility criteria
Perioperative steroid exposure	No standardized structured field	Not modeled	No uniform exposure, timing, or dose field
Baseline comorbidity burden	No standardized index	Not modeled	No cohort-wide Charlson, Elixhauser, or comparable index
Household travel burden	No patient-level measure	Not included in models	No structured data on travel cost, lodging, transportation, caregiver support, or work disruption
Distance to MD Anderson	Available proxy	Included in primary GBM models	Does not represent household travel burden and may be underestimated with temporary local addresses
Access-timing intervals	Not systematically available	Not modeled	Intervals from symptoms to diagnosis, referral, authorization, surgery, radiotherapy, and chemotherapy were not consistently available

Abbreviations: GBM, IDH-wildtype glioblastoma; SVI, Social Vulnerability Index.

Supplementary Table 5. Exploratory astrocytoma subgroup and SVI-group analyses

Analysis	Group	n	Deaths	HR per 0.1 SVI	95% CI	P value	Additional result
Stratum-specific Cox	<40 years	42	18	1.02	0.86-1.21	0.810	
Stratum-specific Cox	40 years or older	20	5	1.09	0.76-1.58	0.638	

Analysis	Group	n	Deaths	HR per 0.1 SVI	95% CI	P value	Additional result
Stratum-specific Cox	Private/commercial insurance	40	15	1.02	0.83-1.26	0.830	
Stratum-specific Cox	Non-private insurance	22	8	1.14	0.86-1.50	0.369	
Interaction	SVI × age 40 years or older	62	23	1.10	0.73-1.64	0.653	No evidence of effect modification
Interaction	SVI × private insurance	62	23	0.84	0.60-1.17	0.302	No evidence of effect modification
Descriptive SVI group	Group 1	10	4				Median OS: 189.5 months; 24-month OS: 100.0%
Descriptive SVI group	Group 2	5	2				Median OS: 19.5 months; 24-month OS: 0.0%
Descriptive SVI group	Group 3	12	4				Median OS: 173.6 months; 24-month OS: 90.9%
Descriptive SVI group	Group 4	35	13				Median OS: 147.6 months; 24-month OS: 87.6%

Abbreviations: CI, confidence interval; HR, hazard ratio; OS, overall survival; SVI, Social Vulnerability Index. Subgroup and interaction analyses were exploratory. Fixed county-SVI groups were <0.25, 0.25-0.49, 0.50-0.74, and ≥0.75. A four-group Cox model was not reported because tied SVI values and sparse events made the estimates unstable; group-specific survival is therefore presented descriptively.

Supplementary Table 6. Proportional-hazards diagnostics and time-varying sensitivity analysis

Model	n	Deaths	SVI HR	95% CI	Cox P	SVI PH P	Global PH P
GBM county primary	395	251	1.00	0.96-1.05	0.920	0.162	0.016
GBM county + RT/TMZ + trial	395	251	1.00	0.95-1.04	0.872	0.137	0.001
GBM tract primary	260	157	1.05	0.98-1.13	0.176	0.051	0.145
GBM tract + RT/TMZ + trial	260	157	1.07	0.99-1.15	0.087	0.066	0.090
Astrocytoma county SVI-only	62	23	1.04	0.89-1.21	0.643	0.661	0.661
Astrocytoma tract SVI-only	39	11	1.12	0.85-1.49	0.422	0.505	0.505
GBM tract extended Cox at 6 months	260	157	1.17	1.04-1.32			
GBM tract extended Cox at 12 months	260	157	1.09	1.01-1.17			
GBM tract extended Cox at 24 months	260	157	1.01	0.92-1.10			

Abbreviations: CI, confidence interval; HR, hazard ratio; PH, proportional hazards; RT, radiotherapy; SVI, Social Vulnerability Index; TMZ, temozolomide. PH P values are Grambsch-Therneau score tests based on Schoenfeld residuals with a left-continuous Kaplan-Meier transformation of time. The GBM tract SVI-by-log-time interaction P value was 0.028.

Supplementary Table 7. Insurance-coding sensitivity analyses

Scale/cohort	n	Deaths	SVI HR per 0.1	95% CI	P value	Excluded categories
GBM county	384	245	1.01	0.96-1.05	0.793	Other and unknown
GBM tract	256	155	1.05	0.98-1.13	0.163	Other and unknown
Astrocytoma county	59	23	1.06	0.90-1.23	0.504	Other
Astrocytoma tract	38	11	1.11	0.83-1.48	0.493	Other

Abbreviations: CI, confidence interval; HR, hazard ratio; SVI, Social Vulnerability Index. Sensitivity analyses excluded other and unknown insurance categories for GBM and the other category for astrocytoma. The source insurance field combined self-pay and uninsured status.
